# Bio-Packaging Material Impact on Blueberries Quality Attributes under Transport and Marketing Conditions

**DOI:** 10.3390/polym13040481

**Published:** 2021-02-03

**Authors:** María Julieta Bof, Franco Emanuel Laurent, Facundo Massolo, Delia Elisa Locaso, Florencia Versino, María Alejandra García

**Affiliations:** 1 Facultad de Ciencias de la Alimentación, Universidad Nacional de Entre Ríos, Monseñor Tavella 1450, Concordia 3200, Entre Ríos, Argentina; julieta.bof@uner.edu.ar (M.J.B.); laurentf@fcal.uner.edu.ar (F.E.L.); locasod@fcal.uner.edu.ar (D.E.L.); 2 CIDCA (Centro de Investigación y Desarrollo en Criotecnología de Alimentos), Facultad Ciencias Exactas Universidad Nacional de La Plata (UNLP)-CONICET La Plata, 47 y 116 S/Nº, La Plata B1900, Buenos Aires, Argentina; facundomassolo@quimica.unlp.edu.ar (F.M.); florencia.versino@ing.unlp.edu.ar (F.V.)

**Keywords:** active packaging, biodegradable polymers, biopolymers, bio-based polymers, natural additives

## Abstract

Blueberries are highly appreciated for their high antioxidant content but are also particularly susceptible to fungal deterioration. In this work, corn starch and chitosan, byproducts of the fishing industry, as well as active compounds obtained from citrus processing waste were used to obtain active biodegradable film packaging. Blueberries were packed in corn starch–chitosan (CS:CH) films and in active films containing lemon essential oil (LEO) or grapefruit seed extract (GSE). The effects of film packaging on the quality parameters of berries and the fungal incidence of disease during storage were studied and compared to benchmark materials. A conservation assay simulating transport and commercialization conditions was conducted. Blueberries packed in CS:CH films showed antioxidant capacity values closer to those packed in commercial PET containers (*Clamshells*), preserving 84.8% of the initial antioxidants content. Fruit packed in LEO films exhibited the greatest weight loss and rot incidence, and poor surface color. CS:CH and GSE films controlled the fruit respiration rate and weight loss, therefore they are materials with adequate barrier properties for blueberries conservation. Bags formulated with GSE showed adequate barrier properties to maintain fruit quality attributes without the incidence of rottenness, being an interesting option for blueberries exportation.

## 1. Introduction

According to the United Nations (UN) on average 13.8% of the food produced worldwide is lost after harvest and during transportation, storage, and processing [1]. Besides aiming to a good balance between food demand and production, adequate packaging systems are necessary to enhance food security and reduce food waste. Packaging materials must be sufficiently resistant to protect and preserve the product from production through transportation and storage until consumption but are discarded usually with little to no reuse [2]. Because of their low cost and density plastic materials are most frequently used for packaging applications. Approximately 42% of the global plastic production is consumed by the packaging sector, being generally disposable items that contribute to the generation of large volumes of waste [3]. Even though the amount of post-consumer plastic waste sent to recycling has more than doubled in the last 15 years (32.5%), almost 25% was still sent to landfill in 2018 [4]. In addition, oil-based plastic materials are basically non-renewable therefore it is well-known that their extensive use contributes to energy source depletion and greenhouse gas emission. Consequently, biobased, biodegradable and compostable plastic materials have been extensively studied as an alternative to reduce waste generation and plastic pollution.

Many bioplastics from biopolymers such as starch, cellulose, and proteins, synthetized plastics from biomass (PLA) or produced by microorganisms (PHAs and PHBs) have been extensively studied as alternative materials for food packaging [5,6,7,8]. Among these, chitosan has been widely used for packaging applications because of its biodegradability, non-toxicity, film-forming properties, chemical stability, and intrinsic antimicrobial and antioxidant properties [9]. Numerous studies in the applications of engineered chitosan-based films in food packaging, including composite films with other biopolymers and active compounds, have been reported and revised [9,10,11,12]. In this regard, active food packaging materials appear to be a promising technology for extending food shelf-life and preserve their nutritional and commercial quality, especially for highly perishable agricultural products [12,13]. Specifically, the addition of natural antimicrobial and antioxidant agents such as plant extracts and essential oils to bioplastic materials provide a more sustainable alternative to conventional synthetic food packaging.

Blueberries (*Vaccinium corymbosum* L.) are recognized as an excellent source of natural antioxidants, which have proven health benefits for prevention and treatment of neurodegenerative and cardiovascular diseases, diabetes, and cancer, among others [14,15,16]. Yet, blueberry is a highly perishable fruit and deteriorates rapidly after reaping, mainly because of its high respiratory rate. Such deterioration is evidenced by the appearance of dehydration, softening, loss of juice through injuries, mold growth, among others that results in non-marketable fruit [17,18].

Consumer preferences, especially in mature markets such as USA, Europe, and Oceania, tend to be shifting toward fresher fruit products, including frozen fruit. In this respect, worldwide blueberry production has shown a marked increase over the last 20 years, reaching 552,000 tons in 2016 [19]. Because of their seasonality, fresh blueberries are alternately produced both in the Northern and Southern Hemisphere. Shipments among distant international markets are made primarily by air or sea. Even though larger fruit volumes are moved by sea because of its lower cost, this means of transport requires several days to reach the destination during which diseases produced by mold such as *Botrytis cinerea* are facilitated [20]. These microorganisms produce spores that resist post-harvest treatments infecting the fruit during transportation with the consequent fruit quality loss. Therefore, the use and development of innovative conservation technologies to prevent, or at least to delay, blueberries quality attributes loss is of great interest. Currently, post-harvest conservation strategies for fresh blueberries consist of single or combined technologies such as modified atmosphere, ozonation, UV radiation, fumigation with SO_2_, and refrigeration temperatures close to the fruit freezing point [21,22,23,24,25]. 

The ideal alternative treatment to control post-harvest diseases should not have any negative influence on the fruit, the environment or human health and should be in accordance with food safety guidelines. In this regard, edible biodegradable coatings and films are interesting alternatives that are currently applied on an industrial scale mainly in citrus and pome fruit processing. Mannozzi et al. [26], Abugoch et al. [27], and Sun et al. [28] have worked on the application of coatings on the skin of blueberries to prolong their post-harvest life with positive results. However, this methodology would not be entirely adequate since the coating modifies the natural waxy coating that covers the skin of the fruit, known as *bloom*, which is an indicator of fruit quality. Color and luminosity changes have been reported by the authors [26,27], indicating that further sensorial properties would be required. Moreover, Chu et al. [29] have found that by removing this natural waxy layer accelerates the deterioration and weight loss processes, thereby reducing the shelf-life as well as the sensory and nutritional attributes of the blueberries.

Alternatively, the use of active packaging, meaning that a substance with a specific function is incorporated to the material to control the product quality and sensory properties by modifying their environmental conditions, could replace costly conventional food processing techniques and prevent fruit surface alterations. Thus, there has been a growing interest in developing antimicrobial and antioxidant packaging materials with natural agents to prevent the growth of foodborne pathogens and microorganisms [30]. Active materials are usually composite materials with the active compound itself or other material particles that contain the active agent. Besides, the use of biodegradable and biobased polymers has been largely studied for low environmental impact packaging materials, constituting an attractive alternative as matrix for green active packaging materials [31,32,33,34]. Among the available natural polymers and compounds, some can be obtained from agro-industrial waste and could be used in value-added applications. In this respect, chitosan is a widely studied biodegradable polymer derived from chitin that can be obtained from fishing industry residues such as crustaceous exoskeletons, which annually account for over 60,000 tons of waste [35]. Besides, it presents promising characteristics for food packaging applications because of its antioxidant and antimicrobial activity, biodegradability, non-toxicity, biocompatibility, film-forming capacity, and chemical stability [9].

Synthetic chemical preservatives commonly used in antimicrobial food packaging include organic acids and their salts, sulfites, chlorides, phosphates, epoxides, hydrogen peroxide, antibiotics, and bacteriocins [3]. However, the use of plant extracts and essential oils (EOs) as additives for active food packaging have been studied and reviewed aiming to replace synthetic preservatives because of their antioxidant and antimicrobial properties and Generally Recognized as Safe (GRAS) character [36,37]. Agroindustrial by-products, particularly from fruit and vegetables processing, contain vitamins, minerals, antioxidants, and antimicrobial compounds for food preservation, though are often discarded or used for animal feed [3]. Thus, the use of these active compounds from agricultural by-products not only contribute to the recovery of these compounds with specific activities but also generate added value to them.

The use of EOs can affect the material microstructure as well as their mechanical and barrier properties [13,37,38,39]. In a previous work, active biodegradable films based on corn starch and chitosan with lemon essential oil (LEO) and grapefruit seed extracts (GSE) were developed and characterized, showing good material properties and antibacterial activity [40]. These oils are also byproducts or residues derived from the citrus processing industry.

The aim of this work is to assess the performance of biodegradable sustainable active packaging with active compounds obtained from fishing and citrus industry processing waste on fresh blueberries preservation. A comparative study between the biobased materials and benchmark synthetic plastic packaging systems, PET *clamshell* containers and modified atmosphere (MA) bags, was conducted. The evolution of the fruit main quality attributes as well as the rot incidence of mold in the packed berries during cold storage and thermal abuse were analyzed, considering typical transport and commercialization conditions of the fresh product. Finally, the correlation among the packaging materials properties and fruit quality parameters was also studied.

## 2. Materials and Methods

### 2.1. Materials

Chitosan of 444 kDa molecular weight and 85% deacetylation degree was provided by Parafarm (Buenos Aires, Argentina). Corn starch with 25% amylose content was purchased from Glutal (Argentina), grapefruit seed extract (GSE) was provided by Euma SAICIYF (Buenos Aires, Argentina), and lemon essential oil (LEO) was supplied by Litoral Citrus S.R.L. (Concordia, Argentina). Acetic acid (99%, analytic grade) was used to solubilize chitosan powder and glycerol (Anedra, Buenos Aires, Argentina) was used as a plasticizer at 25% *w*/*w* in all formulations.

### 2.2. Biodegradable Films Preparation

Active biodegradable films were obtained by casting method from filmogenic suspensions formulated with chitosan and corn starch, which were optimized in a previous work [40]. Briefly, chitosan (CH) was dispersed in aqueous acetic acid to obtain a 2.5% *w*/*w* solution. On the other hand, a 4% *w*/*w* corn starch (CS) suspension was gelatinized at 90 °C for 20 min in a thermostatic bath. Both biopolymers were mixed in a 75:25 CS:CH proportion. Glycerol was added as a plasticizer at 25% *w*/*w*. Two essential oils were chosen because of their antimicrobial activity: lemon essential oil (LEO) and grapefruit seed extract (GSE). The essential oils were added at 3% *w*/*w* in formulations according to a previous work [40]. The film forming dispersions were homogenized in a rotor-stator Ultraturrax T25 (Janke & Kunkel, Staufen im Breisgau, Germany). Then they were poured into rectangular plates of 10 × 15 cm^2^ and dried in a convection oven (FAC, Buenos Aires, Argentina) at 50 °C until constant weight. The films were removed from the plates and thermo-sealed with an impulse sealer (Lepari, Santa Fe, Argentina) to obtain the active biodegradable packaging.

**Figure 1:**
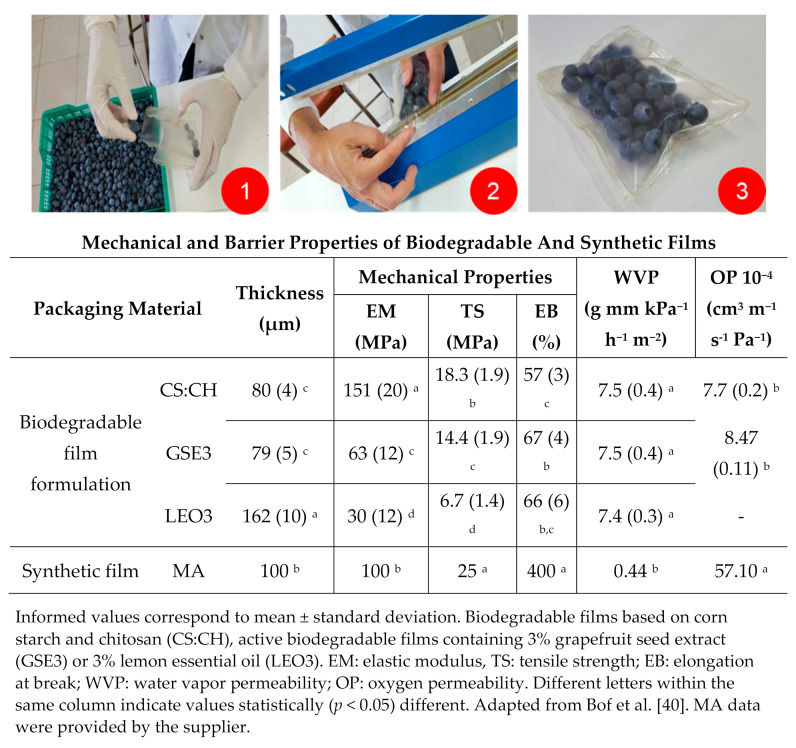
Blueberries packaging procedure using biodegradable active films and their relevant properties.

Synthetic PET containers, commonly called *Clamshells*, (125 g or 4.4 oz capacity, with dimensions of 106 × 106 × 40 mm^3^) and commercial bags suitable for modified atmosphere (DISEVAC SS MB Cristal 150 × 200, Plásticos DISE S.A., Córdoba, Argentina) were used as benchmark packaging systems. Barrier and mechanical properties of flexible film materials are included in Figure 1.

### 2.3. Blueberries Preservation Assays under Transport and Market Conditions

In order to determine the effects of the active biodegradable packaging on the main quality attributes of commercial fresh blueberries under refrigeration condition, fresh Emerald blueberries cultivated in Salto Grande (31°28′13.746′′ S, 58°9′10.929′′ W) were used. Fruit was harvested with an optimum maturity degree, selected by size, shape, and color, discarding the damaged or altered fruit. The selected blueberries were packed in five different containers: 75:25 corn starch-chitosan biodegradable film (CS:CH); biodegradable active film with 3% GSE (GSE3); biodegradable active film with 3% LEO (LEO3); commercial bags for modified atmosphere (MA) and PET *Clamshells* (CL).

All containers were filled with 25 berries each (approximate weight: 55 g), and both biodegradable films and MA commercial bags were sealed. In this case, 5 replications were made for each treatment. A batch of 1.5 kg of blueberries was used in this experiment.

Ship transport conditions simulation assays for blueberries exportation were conducted in a cooling chamber with controlled temperature and relative humidity (1 ± 0.5 °C and RH > 85%, respectively) during 30 days. Subsequently, samples were kept for 7 days at 20 °C to simulate shelf storage and marketing conditions. The fruit quality attributes were analyzed throughout the whole storage period.

### 2.4. Quality Attributes of Packed Fruit

#### 2.4.1. Weight Loss

Fruit weight loss was determined at the end of cold storage and after 7 days of storage at 20 °C. An OHAUS SP 602 scale (New York, NY, USA) was used and the result was expressed as percentage of weight loss with respect to the initial sample weight. The informed values correspond to the average of ten replicates.

#### 2.4.2. Firmness

Blueberries firmness tests are based on skin toughness measurements considering puncture force and penetration or deformation of the fruit. Several authors have commented on the reproducibility of puncture tests thus a similar probe than that used by other authors was selected for the puncture tests [41,42,43]. Firmness was determined as the puncture resistance using a texturometer (TA.XT2i Stable Micro System, Godalming, UK) with a 2 mm cylindrical probe (P/2) at a rate of 1.0 mm/s. The fruit was penetrated by the probe in the equatorial zone. In order to minimize the variability, the informed values correspond to the mean of 30 randomly selected blueberries.

#### 2.4.3. Color

Surface color was evaluated using a Minolta CR-300 colorimeter (Osaka, Japan) with D65 illuminant on the CIELab color scale. The value of L represents the luminosity and varies from 0 (black) to 100 (white); the parameter a* indicates green (−) to red (+), while b* varies from blue (−) to yellow (+). Three measurements per fruit were performed and the informed values correspond to the mean of 25 fruit per sample, at the beginning and at the end of the test period. Color differences (ΔE) were calculated as described in a previous work [44] with respect to the initial color parameters values.

#### 2.4.4. Titratable Acidity and Total Soluble Solids

The titratable acidity (TA) was measured by potentiometric titration at pH 8.3 with a pH-meter (Hanna, Woonsocket, RI, USA) applying the method 942.15 of the Association of Official Analytical Chemists (AOAC, 2005). Considering that the main organic acids present in blueberries are citric and succinic acid, exhibiting higher content of the first one, the titratable acidity was expressed as citric acid content per 100 g of fruit (g citric acid per 100 g fruit).

Total soluble solids (TSS, %), were determined by the refractometric method (AOAC 983.17–976.20, 1990), using a digital Abbe type refractometer, with temperature compensator (Misco, St Paul, MN, USA).

For each determination, three samples of 5 g from a lot of 25 blueberries randomly selected at the beginning and the end of storage time were analyzed.

#### 2.4.5. Respiration Rate

The respiration rate of the fruit was determined by a quasi-stationary state method placing 120 g of fruit in a 1.5 L capacity glass container. This was hermetically sealed and connected by a septum to a CheckMate 3 Dansensor gas meter (Ringsted, Denmark). The equipment has a zirconium sensor for O_2_ measurement and a non-dispersive infrared CO_2_ sensor. The gases concentration (%) inside the glass jars was measured every hour for 8 h at 25 °C. CO_2_ and O_2_ percentages were plotted as a function of time and data were linearly regressed. According to Fonseca et al. [45], the respiration rate was expressed in mL kg^−1^ h^−1^ and calculated in terms of CO_2_ produced and O_2_ consumed as follows:

RO_2_ = V × sO_2_/w (1)

RCO_2_ = V × sCO_2_/w (2)

where sO_2_ and sCO_2_ are the slopes corresponding to the regression lines of O_2_ and CO_2_ percentage concentration as a function of time (h), w (kg) is the fruit weight, and V (mL) is the available headspace volume in the container.

In addition, the respiratory quotient (RQ) was determined as the proportion of CO_2_ produced to O_2_ consumed by the product. Tests were carried out, at least in duplicate, at the beginning and at the end of the storage time.

#### 2.4.6. Antioxidant Activity

Blueberries are valued nutritionally for their high content of antioxidants [46]. Thus, to evaluate the losses of these compounds during storage the antioxidant activity of the blueberries was determined at the initial and final time using the ABTS technique. In addition, the total phenolic compounds content was measured by the Folin-Ciocalteu method.

First, ethanolic extracts were prepared. Pulp tissue frozen with liquid nitrogen was ground to a powder in a grinder (Ultracomb 8100a, Buenos Aires, Argentina). Approximately 0.1 g of sample were weighed and diluted with 5 mL of ethanol (Porta, Buenos Aires, Argentina). The suspension was vortexed and centrifuged at 12,000× *g* for 10 min at 4 °C in a Rolco 2036 centrifuge (Rolco, Buenos Aires, Argentina).

To quantify the antioxidant capacity (AC), a solution was prepared with 0.0192 g of the ABTS reagent (MW = 548.68 g mol^−1^) and 0.0033 g of K_2_S_2_O_8_ (MW = 270 g mol^−1^) to a final concentration of 7 mM ABTS and 2.45 mM of K_2_S_2_O_8_. A total of 5 mL of this solution was stored in dark at room temperature for 12 to 16 h without agitation to favor the formation of the ABTS *+ radical. Then, an aliquot of the solution was taken and diluted with ethanol until an absorbance of 0.7 ± 0.05 at 734 nm was reached. Subsequently, 50 μL of the ethanolic extracts of the samples and 1 mL of the ABTS *+ radical solution prepared above were mixed under constant agitation and after 6 min the absorbance at 734 nm was measured in a spectrophotometer (UV-Mini Hitachi, New York, NY, USA). As blank control sample, 50 μL of ethanol were placed with 1 mL of the ABTS *+ solution. For the measurements to be reliable, the percentage of reduction of the absorbance must be between 20 and 80% with respect to the target. The calibration curve was performed with Trolox reagent (Sigma-Aldrich, St. Louis, MO, USA), a water-soluble analog of α-tocopherol, as standard. The determinations were made at least in triplicate using independent extracts. The results were expressed as Trolox equivalent antioxidant capacity (TEAC) by fresh weight in mg kg^−1^.

Likewise, the content of total phenolic compounds (TPC) was quantified using the Folin-Ciocalteu technique. For each sample, 150 μL of fresh tissue ethanolic extract were placed in tubes with distilled water (until 1350 μL total volume) and 50 μL of 1 eq L^−1^ of Folin-Ciocalteu reagent (Sigma-Aldrich, St. Louis, MO, USA). The samples were homogenized by vortexing for 3 min at 20 °C before 100 μL of 20% Na_2_CO_3_ (*w*/*v*) in 0.1 eq L^−1^ of NaOH was added. The tubes were vortexed and incubated at 20 °C for 1 h in a dark place. Absorbance at 760 nm was measured in a spectrophotometer (UV Mini-1240, Shimadzu Corporation, Tokyo, Japan). For quantification, a calibration curve was made with chlorogenic acid (Sigma-Aldrich, St. Louis, MO, USA), the predominant phenolic compound in blueberries, using a standard solution of 224 μg/mL. Samples were measured at least in triplicate using independent extracts. The results were expressed as equivalent milligrams of chlorogenic acid per kilogram of fresh weight (mg kg^−1^).

#### 2.4.7. Fungal Decay

The post-harvest infection of the fruit at the end of the refrigerated storage was evaluated by macroscopic observation. The first visible infection point was counted as indicative of rot and the result was expressed as a percentage of deteriorated fruit with respect to the total analyzed [47,48]. Lots of 1.5 kg fruit were evaluated.

### 2.5. Statistical Analysis

The obtained results were statistically analyzed using the Statgraphics Plus software for Windows 5.1 (Manugistics Corp., Rockville, MD, USA) performing the analysis of variance (ANOVA). Likewise, the means comparison was carried out using the Fisher’s minimum difference test (LSD) with a confidence level of 95%. Results were subjected to principal components analysis (PCA) and cluster analysis (CA). Data were analyzed using the Infostat software v2011 [49].

## 3. Results and Discussion

### 3.1. Blueberries Quality Attributes

**Figure 2:**
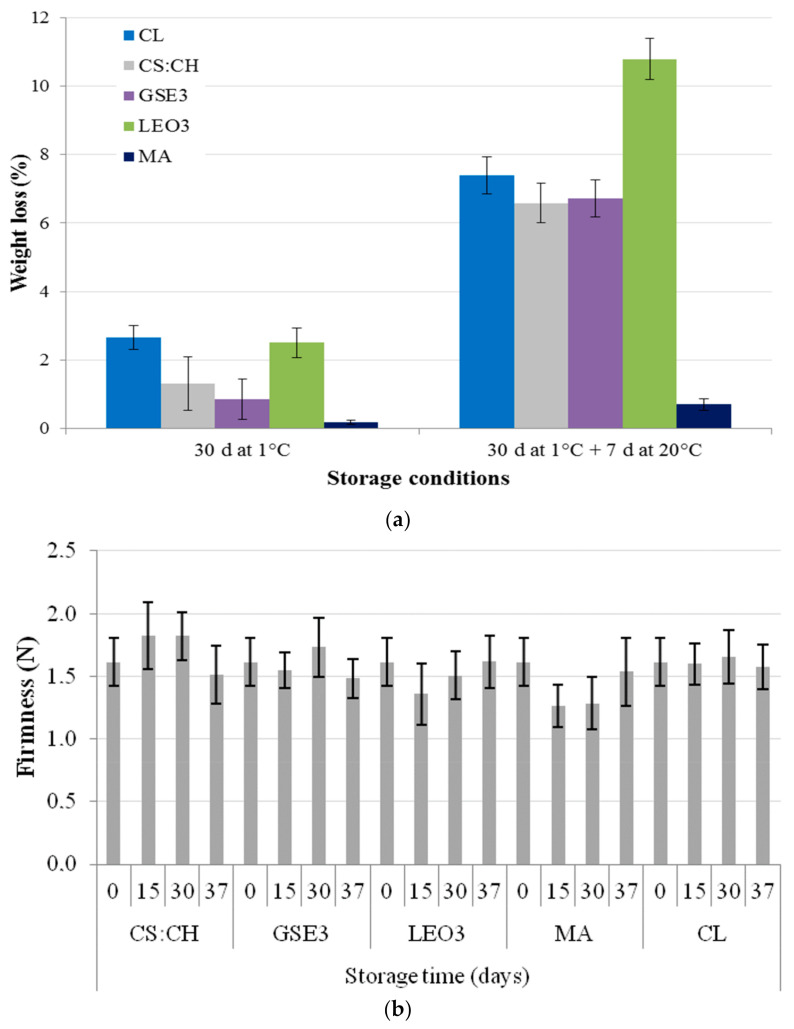
(**a**) Weight loss and (**b**) firmness of blueberries packed in clamshell (CL), biodegradable films based on corn starch and chitosan (CS:CH), active biodegradable films containing 3% grapefruit seed extract (GSE3) or 3% lemon essential oil (LEO3), and modified atmosphere commercial bags (MA). Blueberries were stored for 30 days under refrigeration conditions and then kept for 7 days at room temperature.

The evolution of weight loss of blueberries after the two storage stages is shown in Figure 2a. Among the fruit packed with biodegradable films, blueberries in LEO3 bags exhibited the higher weight loss. These results correlate with the microstructure of the active films containing LEO studied in a previous work [40] and its negative impact on water vapor barrier properties, which ultimately determine the product dehydration. In a previous work, an exhaustive microstructural characterization of active films containing GSE and LEO was performed [40]. SEM analysis evidenced that LEO presented marked defects (visualized as oil micro-droplets) because of the lack of polymer miscibility, which introduced a great number of discontinuities in the matrix. Similar results were found by do Evangelho et al. [39] who incorporated orange essential oil in starch-based films and informed the presence of pores in the film cross-section that facilitated the passage of water vapor and consequently increased WVP. Discontinuities and pores in film matrix evidenced by microstructural analysis were also reported by Sánchez-González et al. [38] working with tea-tree essential oil and Atarés et al. [50] using ginger oil.

Blueberries packed with CS:CH and GSE3 films exhibited lower weight losses than the control (*Clamshell*, CL) (*p* < 0.05). In contrast, the samples packed in active films LEO3 had weight losses similar to CL, since they were inefficient barriers to water vapor during the first storage stage. Moreover, when fruit was transferred to room temperature after 30 days of refrigerated storage samples packed in active films LEO3 exhibited the highest weight loss, probably due to structural defects induced in the composite films by the thermal shock that could affect WVP of films.

The lowest weight loss was observed for fruit contained in MA (Figure 2a). This result could be explained considering both fruit respiratory rate [51] and synthetic film low water vapor permeability (Figure 1). Internal gas modification in MA packaging, reduces the fruit respiration activity, which consequently reduces transpiration rate, therefore reducing weight loss [52]. Concha-Meyer et al. [42] found similar values of weight loss for blueberries preserved in modified atmospheres.

As a result of dehydration, superficial wrinkling occurs which makes fruit appearance less attractive. This withering is the result of cellular plasmolysis that is evidenced when fruit moisture contents losses are greater than 5–10%. It has been suggested that the maximum weight loss for blueberries before they lose their commercial quality should be 5–8% [53,54]. Considering a limit value of acceptability of 8% for this parameter, the shelf life of packaged fruit would be acceptable after 30 days of storage in all cases. Meanwhile, under simulated thermal abuse conditions (30 days at 1 °C and 7 days at 20 °C) that would occur at the sales points, with the exception of those containing LEO, all containers would meet this quality standard. These results indicate that these biodegradable containers could be used for the exportation of blueberries and their transportation under refrigeration conditions.

On the other hand, no significant differences were found (*p* > 0.05) in firmness among all samples (Figure 2b), therefore the fruit maintained its physical characteristics during the conservation period at 1 ± 0.5 °C and 90% RH. Moreover, there were no significant (*p* > 0.05) changes in firmness during thermal abuse (indicated as 37 in Figure 2b). The obtained results are in agreement with those reported by Chiabrando et al. [55] who stressed that firmness was not a critical quality factor. Besides, low temperature storage conditions slow down the fruit softening process by inhibiting enzymatic activity and ethylene production [42].

It should be remarked that the high standard deviations observed for the informed values (Figure 2b) are attributed to differences in the fruit size, since smaller blueberries tend to be a little firmer than the larger ones, giving an inversely proportional relationship between size and firmness for the same variety [22,56,57].

Mean value differences observed in firmness of LEO3 packed fruit after 15 days could be due to the weight loss during storage that leads to less turgid berries, as has been reported for various crop varieties [58,59]. Fruit shriveling makes tissues more rubbery, thus when the probe exerts pressure on the fruit during the puncture test the peel deforms more before the surface tension is exceeded and the irreversible rupture occurs [60]. Likewise, fruit softening observed for MA samples could be attributed to respiration conditions differences under cold and ambient temperature conditions [52,61].

**Figure 3:**
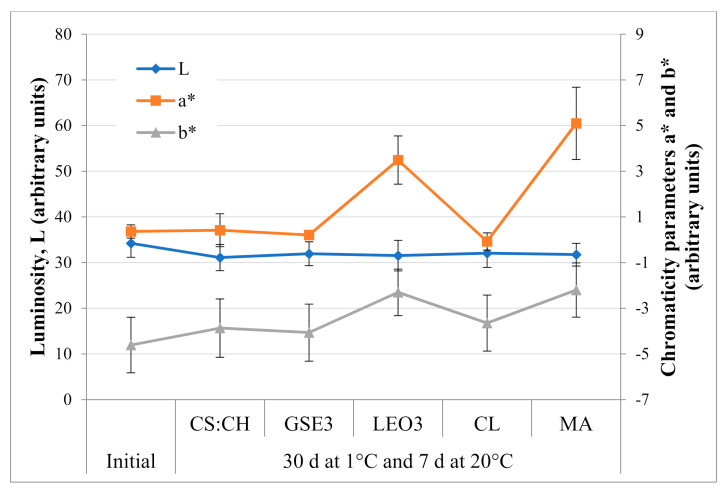
Evolution of surface color parameters of blueberries Emerald var. during refrigerated storage. Fruit were packed in clamshell (CL), biodegradable films based on corn starch and chitosan (CS:CH), active biodegradable films containing 3% grapefruit seed extract (GSE3) or 3% lemon essential oil (LEO3), and modified atmosphere commercial bags (MA).

Regarding surface color parameters, it has been reported that the luminosity (L) and chromaticity parameter (b*) values can be affected by the natural waxy layer covering the berries surface known as *bloom* [45,46]. The results of the surface color evolution before and after storage are shown in Figure 3. No significant changes (*p* < 0.05) were observed in the L and b* parameters, except for the fruit packed with LEO active films and MA. This was also evidenced through the color differences (ΔE) calculated with respect to the initial values. The ΔE values obtained were 2.39 ± 0.11, 3.21 ± 0.46, and 2.35 ± 0.44 for the blueberries packed in *clamshells*, flexible biodegradable bags of CS:CH and the active ones containing 3% GSE (GSE3), without significant differences among them (*p* > 0.05). On the other hand, fruit packed in the active flexible bags with LEO at 3% and those packed in the bags for modified atmosphere (MA) presented statistically higher (*p* < 0.05) ΔE values, 4.73 ± 0.84 and 5.86 ± 1.40 respectively, without significant differences between them.

Blueberries *bloom* contains various lipidic components, mainly triterpenoids and diketones [29]. Its main function is to protect the fruit against external agents, prevent its dehydration and softening, among others. Besides, the lemon essential oil used in LEO3 films contains compounds of a lipophilic nature, limonene being the most important. As it was reported in a previous work, LEO was not efficiently incorporated into the composite polymeric matrix [40]. Therefore, surface migration of the active compound could occur affecting the waxy layer of the fruit. In this regard, blueberries stored in LEO3 bags had lost their characteristic waxy appearance. This would explain the LEO3 low efficiency in preserving fruit moisture and color. Thus, the observed high fruit weight losses (Figure 2) could be attributed both to the matrix discontinuities that LEO introduces in the polymer matrix and the loss of the fruit protective waxy layer.

Furthermore, Perdonés et al. [62] indicated that the presence of LEO in the formulation of coatings based on chitosan for strawberries affected the fruit metabolism modifying the breathing patterns, the enzymatic activity and the physiological maturity, which is evidenced by changes in the color attributes of the fruit. Yet, the use of edible films on blueberries is still not commercially feasible for two main reasons: First, there are technical adherence problems due to the smooth waxy skin of the fruit; second, the coated fruit takes an artificial glow that renders the fruit unattractive.

With respect to the fungal decay, the packed Emerald blueberries did not show mold growth at the end of the refrigerated storage period (30 d). However, when fruit was exposed at room temperature for 7 days the microorganisms present in the fruit manifested (Table 1). The best results were obtained with the MA and GSE3 films, however blueberries packed in CS:CH bags also had a protective effect against the microorganisms that deteriorate the fruit compared to control fruit packaging (CL). Samples packed in LEO3 had a significantly higher percentage of rot incidence than the others, that correlates with the changes in the other quality attributes previously analyzed.

### 3.2. Fruit Internal Quality

**Figure 4:**
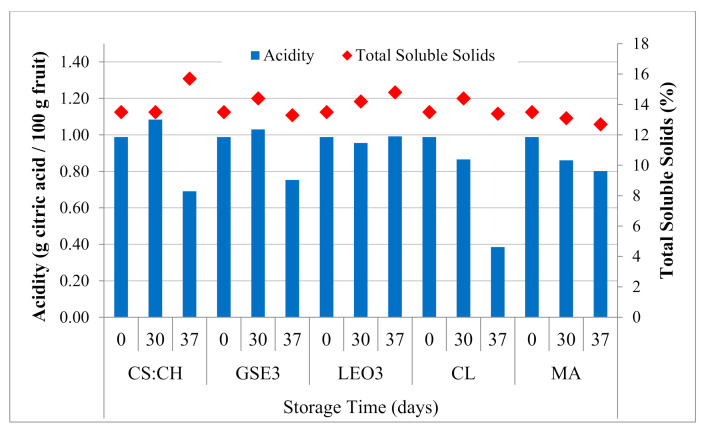
Evolution of titratable acidity and total soluble solids of blueberries Emerald var. during refrigerated storage. Fruit were packed in clamshell (CL), biodegradable films based on corn starch and chitosan (CS:CH), active biodegradable films containing 3% grapefruit seed extract (GSE3) or 3% lemon essential oil (LEO3), and modified atmosphere commercial bags (MA).

Blueberries were harvested with pH = 2.57, TSS = 13.5%, TA = 0.98 g citric acid per 100 g fresh fruit. Blueberries packed in biodegradable (CS:CH) and active films (GSE3 and LEO3) maintained the maturity index during the storage under refrigeration condition, since no significant (*p* > 0.05) variations were found in the ratio between the TSS and TA of the fruit. Contrastingly, fruit packed in commercial materials (*clamshell*, CL, and synthetic film for modified atmosphere, MA), showed a significant (*p* < 0.05) decrease in TA (Figure 4). When samples were transferred to room temperature and stored for 7 days (indicated in Figure 4 as 37) all samples increased the maturity index evidenced by the decrease in TA. Similar results were reported by Harb and Streif [63] and Almenar et al. [54]. However, the detriment was more evident in CL packed fruit, indicating that, both the biodegradable and active films and the MA commercial bag were more efficient in delaying the fruit senescence.

Blueberries are considered climacteric fruit, since their respiratory rate increase twice during its development: one with the beginning of the coloration where a transition from the green-pink to a blue-pink state occurs and a second one when the over-ripening begins [64]. Anthocyanins located in the skin and the pulp are responsible for the blue coloration of the fruit, being the main pigment malvidin. The color of the skin is used as a harvest indicator, and once the state is completely blue, the color does not change, whereas acidity, soluble solids, and pH do [65].

Inside the active films packaging and the MA synthetic film an atmosphere equilibrium is reached between the blueberries respiratory activity and the gas permeability of the packaging material. In contrast, this balance would not be given in the benchmark *clamshell* packages because of the continuous ventilation that these containers allow.

The evolution of the blueberry respiratory rate of the Emerald variety is presented in Table 1. All the containers were effective in controlling the respiratory rate of the fruit during refrigerated storage. As expected, the smallest variation in the respiratory quotient (RQ) was obtained for synthetic films for modified atmosphere with proven differential gas barrier capacity. TheRQ is the proportion of CO_2_ produced to O_2_ consumed by the product and its value ranges between 0.7 and 1.3 in aerobic respiration depending on the metabolic substrate [66]. Beaudry et al. [67] reported that blueberries normally have respiratory quotients of 1.3 because of the high content of citric acid and sugars. According to this criterion the fruit packed with MA commercial bags presented anaerobic respiration conditions, which would render them unsuitable for this product. Changes in fruit color (Figure 3) and firmness (Figure 2) could be attributed to the observed respiratory conditions during storage. Such alterations in respiration rate can occur due to temperature changes resulting in variations in gas ratio and moisture conditions within the bag [52,61]. Meanwhile, the RQ values of the control packed in CL and those using the biodegradable films are within the acceptability limit for this criterion.

Another novel approach to fruit preservation is the use of biodegradable containers and edible coatings based on different biopolymers containing active compounds that has been widely reported in the literature [26,27,50,68,69,70,71,72,73,74]. In this regard, Perdonés et al. [62] studied the behavior of strawberries coated with chitosan and lemon essential oil. These authors found that lemon oil affected the metabolism of strawberries by modifying the respiration patterns of the fruit. The RQ also increased and volatile compounds were detected in the fruit, related to fermentative metabolism (acetaldehyde and ethanol) indicating anaerobiosis conditions. These modifications were attributed to cell interactions with chitosan or essential oil that induced cellular stress and changes in the enzymatic activity of the fruit. Nonetheless, as it was previously remarked, the use of edible coating is not commercially viable for blueberries, because the superficial *bloom* is lost during the coating procedure application.

On the other hand, Almenar et al. [75] found that when blueberries are packed in biodegradable PLA (polylactic acid) containers a condition of equilibrium is reached in the headspace after three days of storage. These authors also observed that the composition of gases within the containers depends on the storage temperature. Giuggioli et al. [76] also worked with blueberries packed in biodegradable films and observed that the variations in the respiratory rate during 16 days of refrigerated storage were small, but differences were significant when the containers were exposed to room temperature.

Among the biodegradable films developed in the present work, the lowest RQs were obtained with the biodegradable packaging CS:CH followed by GSE3. Therefore, it can be inferred that these materials have adequate barrier properties for the conservation of blueberries since they showed to have the slightest weight loss during the refrigerated storage (Figure 2a) and a good control of the maturity physiological parameters (Figure 4).

In berries, the most important group of phenolic compounds is the flavonoids, which consist mainly of anthocyanidins, flavonols, proanthocyanidins (condensed tannins), flavones, and their glycosides. Other phenolic compounds present in berries are hydrolysable tannins, phenolic acids, lignins, among others [77,78,79].

Table 2 presents the results of the total phenolic content (TPC) and the antioxidant capacity (AC) after 30 days of storage at 1 °C and 7 days at 20 °C. It is important to note that in the present work it was determined that chlorogenic acid was the main phenolic compound present in blueberries by a preliminary analysis of the ethanolic extracts using reverse phase HPLC, hence the results were quantified with this standard. The obtained TPC values are similar to those reported by Vázquez-Castilla et al. [80], between 414 ± 31.90 and 726.9 ± 66 mg gallic acid/100 g fresh weight, even though the authors expressed TPC using gallic acid as standard. Other authors informed lower TPC than those reported in Table 2 [81,82], though differences can be attributed to the used extraction conditions. TPC and antioxidant capacity were not determined in fruit packed in LEO3 films, considering these determinations irrelevant because of their high dehydration that limits their shelf-life.

Cantín et al. [24] and Giuggioli et al. [76] reported that changes in total phenols content occurred more slowly when conservation temperature was 1 °C, as in the present work.

The antioxidant capacity correlated satisfactorily with the total phenolic compounds content (r^2^ > 0.99). As it was expected, both the TPC and AC decreased, indicating as loss percentage for each parameter in Table 2. Although the AC decreased in all samples, blueberries packed in CS:CH presented a final value closer to that of the fruit in CL followed by samples packed with films containing GSE3. Differences observed between the biodegradable films and the active ones containing GSE3 are noticeable given that they have similar O_2_ permeabilities and that the active films did not show any antioxidant activity per se [40]. Although pure GSE and LEO exhibited antioxidant capacity evaluated through ABTS.+ (2,2-azino-bis(3-ethylbenzothiazoline-6-sulphonic acid)) technique, their concentrations in active films were insufficient to confer this capacity to the developed materials. Nevertheless, in the selection of the active compound content a delicate balance between the antioxidant-antimicrobial capacity and the enhancement in both mechanical and barrier properties of developed material should be carefully considered.

The lower values of both AC and TPC content were obtained for fruit packed with benchmark films suitable for modified atmospheres (MA). Accordingly, Remberg et al. [83] stated that blueberries packed in *clamshells* and preserved in the air presented greater antioxidant capacity than those found in modified atmospheres, which correlates with the present results.

### 3.3. Materials Properties Effect on Fruit Quality Parameters

Both CS and CH are polysaccharides that, being hydrophilic in nature, present poor barrier properties with relatively high values of solubility and permeability to water vapor [84]. This behavior is explained considering the affinity of the composite films for water molecules because of the presence of a large number of hydroxyl groups in the structure of both biopolymers. This causes a weakening of the intra and intermolecular bonds (plasticizing effect) in the presence of humidity, which leads to an increase in the permeability of this type of polymers [85,86]. Even though the addition of LEO was expected to improve the lipophilic/hydrophilic balance of the material and consequently enhance its water vapor barrier properties, its poor miscibility with the polymer matrix induced microstructural defects which resulted in higher weight loss of the blueberries packed in LEO3 bags. The effects of low temperatures and changes in temperature conditions on the materials microstructure and barrier properties should also be considered in this analysis.

Although the same molding ratio was used to obtain active films, thickness was significantly (*p* < 0.05) different because of the variations in the total solid content of the formulations (Figure 1). GSE addition did not notably affect film thickness because of the low solid content of the GSE extract, while LEO led to thicker ones because of the greater amount of the poured solids in the casting plate [40].

Moreover, the compatibility of the active agent with the matrix determines how efficiently the former is incorporated into the polymer network and so the film microstructure characteristics. Consequently, the hydrophilic nature GSE ethanolic extract makes it more compatible with the CS:CH matrix, leading to a more compact and denser film structure. On the contrary, LEO is clearly hydrophobic in nature resulting in more heterogeneous materials with evidence of oil droplets embedded in the polymer blend evidenced by SEM in a previous work [40], which also explains their greater thickness.

The thickness of a packaging material mainly affects its WVP, mechanical resistance, and optical characteristics (transparency), among others. In addition, thickness may alter optimal heat-sealing conditions (time and temperature) used in manufacturing containers. However, no modification of the sealing conditions were needed for LEO3 film bags.

**Figure 5:**
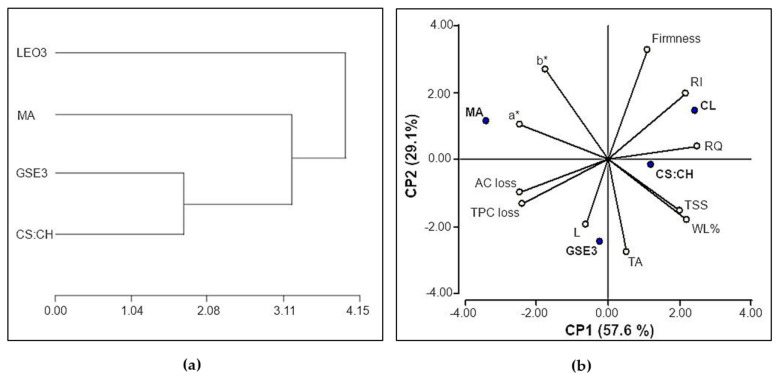
(**a**) Conglomerates analysis of the relevant properties of the materials used for fruit packaging. (**b**) PCA bi-plot (first and second components) of packaging materials’ samples (blue dots) regarding blueberries quality attributes (white dots).

The conglomerate analysis indicated that the mechanical and barrier properties were similar between the biodegradable films: CS: CH and GSE3 active film (Figure 5a). The differences with the synthetic material lie basically in that although it exhibits the lowest WVP, its permeability to oxygen is higher than biodegradable films and presents the best mechanical properties (Figure 1), as expected since it is a multilaminate material with an external LDPE layer. The poor barrier properties of LEO3 biodegradable film explain its difference with the rest of the materials tested. It is important to mention that oxygen permeability of films containing LEO could not be measured because of equipment restrictions [40]. *Clamshells* were omitted in this comparison since they are perforated containers and no barrier properties values could be assigned to them.

Considering the poor performance of LEO3 films on blueberries preservation assay, these samples were not included in the principal component analysis (PCA). This analysis shows that the first two principal components (PC1 and PC2) explained the 86.7% of the total variance with a cophenetic coefficient of 0.982 (Figure 5b). PC1 was associated with the barrier properties of the packaging material (mainly with the oxygen permeability), while PC2 grouped materials according to their source and biodegradability, respectively explaining 57.6% and 29.1% of the total variance. Thus, positive values of PC1 allow to relate blueberries quality parameters like maturity index (TSS and TA), respiratory quotient (RQ), and those related to water vapor permeability of the packaging such as firmness, weight loss (WL%), and rot incidence (RI). Meanwhile negative values of PC1 grouped fruit quality attributes regulated by changes associated with the fruit senescence such as TPC and AC losses as well as those related to surface color chromaticity parameters (a * and b). Samples packed in MA bags presented greater negative impact in this regard, since the modified atmosphere is derived in fruit anaerobiosis (Table 1). As could be expected, this extreme condition affected the physiological parameters of the fruit (TSS and acidity) and possibly the content of anthocyanins that was evidenced in the alteration of the superficial color of the fruit. On the contrary, blueberries packed in CL showed no modifications in respiratory conditions, hence better results in this respect.

Regarding the nature of the packaging material, positive values of PC2 related fruit attributes that conditioned its shelf-life such as RI, RQ, firmness, and chromaticity parameters (Figure 5b). Furthermore, negative values of PC2 grouped physiological quality attributes (TPC and AC losses, TSS and TA) as well as WL%s and luminosity parameter (L).

In summary, blueberries packed in flexible bags with synthetic films (MA) presented a distinctive behavior in the physiological quality parameters that negatively impact the attributes that limit the fruit shelf-life. Flexible bags obtained by heat-sealing active biodegradable films are a viable alternative to the use of *clamshells*, allowing to maintain the quality attributes of blueberries and to reduce the rot incidence with the additional advantage of the biodegradable nature of the proposed system.

## 4. Practical Applications and Future Research Perspectives

The development of more sustainable materials for the packaging industry comprises a more holistic approach to consumer good production systems, tending to a circular economy. Driven by a global environmental awareness, biobased and biodegradable materials aiming to waste generation reduction, climate change prevention, and nonrenewable energy sources use minimization are key players. Chitosan and corn starch-based bio-packaging active films with GSE are fully biobased materials mostly derived from agricultural waste that successfully preserved blueberries under transport and market conditions for 37 days. The renewable, biodegradable, and non-toxic character of the raw materials are positive features that could contribute to a low environmental impact by reducing food spoilage and plastic waste in landfills, reducing greenhouse gases emissions, providing added value to agricultural by-products, and lessening petroleum resources depletion. Nonetheless, a life cycle analysis (LCA) of the herein studied materials is recommended for future research since some biopolymers and biobased packaging systems have shown poorer environmental impact results than their plastic counterparts depending on the assumptions made and the selected application of the materials [87,88]. Careful consideration of the geographical location of the raw materials and package production, as well as the differential processing techniques and end-of-life scenario for each type of packaging are required. For instance, PET *clamshells* may be recycled and incinerated for energy recovery, while composability should be preferred for biodegradable active films depending on the waste management systems applied.

The economic constraints in the production of biodegradable films based on CS:CH and active agents is an important challenge in the large-scale development of such active packaging. On the one hand, regarding the process scale up of the active films obtained on a laboratory scale by casting the film forming dispersions, the simplest technique is the use of tape-casting. However, to meet market demands the use of industrial scale technologies adapting available equipment for synthetic materials, such as extrusion or compression molding is required [11]. In these cases, it would be necessary to protect the active agents because of their thermolability, encapsulation being a viable alternative for this purpose [3]. New technologies such as the formulation of nanoemulsions and the encapsulation of active principles could be useful for this purpose, though further studies are needed in this regard. Likewise, the incorporation of encapsulated essential oils would also allow modulating the release of active compounds and sustaining it over time, which will not only impact the performance of the active packaging to extend the product quality, but also reduce the amount of active agent incorporated into the formulation [37].

On the other hand, the cost of essential oils and production along with the complicated processing steps make it less attractive for food packaging. As regards the production cost, raw materials market prices were considered to estimate CS:CH and GSE3 films cost: $0.8/kg for CS [89], $76.95/kg for MMW CH [90] and $1.98/kg for glycerol [91]; and 86.16/L $ for GSE [92]. Considering the used molding ratio, 2.36 kg of film forming dispersion are needed to obtain 1 m^2^ of film. In view of the filmogenic dispersions composition and molding ratio, the minimum cost in raw materials corresponding to CS:CH films would be 4.59 $/m^2^ while for GSE3 films it rises to 10.68 $/m^2^, due to the active compound inclusion.

Lastly, regarding the legal considerations related to the approval of containers in contact with food, it is important to note that the active ingredients used have GRAS status.

## 5. Conclusions

The present study demonstrated that the biodegradable films based on corn starch and chitosan and the active film containing GSE reduced the post-harvest weight loss of packed blueberries during their refrigerated storage in comparison to CL PET containers. In addition, in contrast to MA plastic bags, all the biodegradable packaging materials were effective in controlling the fruit respiratory rate during storage without changes in respiratory patterns. Although the antioxidant capacity decreased during storage, blueberries packed in CS:CH biodegradable films showed values closer to those packed in CL, the losses being, with respect to the initial content, 15.2 and 5.6% respectively.

Biodegradable bags, except those with LEO3, showed reduced blueberries rot incidence compared to those packed in the commercial containers (CL).

The conservation assay aimed to simulate the real conditions under which blueberries are transported until displayed on shelves for consumers. Such conditions are crucial for proper fruit preservation contemplating that blueberries are high nutritional and valued food that are seasonally exported from one hemisphere to the other having to undergo large periods of time under refrigerated conditions before reaching the market. Considering a limit weight loss value acceptability of 8%, the shelf-life of packed fruits would be acceptable after 30 days of storage in all cases. However, simulating the thermal abuse that would occur at the commercialization points (30 days at 1 °C and 7 days at 20 °C) the containers containing LEO would not meet this quality criterion. Besides, if the criteria for estimating shelf-life is restricted to the absence of rot, only active formulations containing GSE comply with this requirement throughout the analyzed period. Although MA packaging also presented low rot incidence after storage, it has proven to alter the respiratory conditions of the fruit leading to anaerobiosis with significant detriment of the fruit antioxidant capacity.

Overall, despite the limitations presented by the biodegradable materials herein studied, these are valuable in light of developing more sustainable packaging materials for organic products, especially considering that these are biobased and biodegradable materials derived mostly from food industry waste compounds. As we have previously argued, CS:CH films present similar and improved characteristics in preserving blueberries quality attributes in comparison with CL benchmark containers, while GSE3 would be a promising alternative to MA films. Future research should consider the potential effects of using a combination of materials to design bio-packaging systems with improved outcomes.

The results obtained in this work serve as a starting point for the development of biodegradable packaging for highly perishable products. This is an integral research that contemplates not only the use of active biodegradable bags but also evaluates their performance under real conditions of transport and commercialization, also considering the costs and possible scaling of the process.

## Figures and Tables

**Table 1:** Respiration rates (CO_2_ and O_2_), respiratory quotient at 25 °C (RQ) and rot incidence (RI) of Emerald blueberries before (initial) and after 30 days of refrigerated storage for different packaging systems.

Sample	RCO2 (mL kg^−1^ h^−1^)	RO2 (mL kg^−1^ h^−1^)	RQ	RI
Initial	1.31 ± 0.07 ^d^	1.05 ± 0.05 ^d^	1.25	-
CS:CH	6.30 ± 0.32 ^b^	6.06 ± 0.30 ^b^	1.04	12
GSE3	12.87 ± 0.65 ^a^	12.98 ± 0.65 ^a^	0.99	0
LEO3	5.84 ± 0.29 ^b^	4.96 ± 0.25 ^b,c^	1.18	28
CL	2.82 ± 0.14 ^c^	2.09 ± 0.10 ^d^	1.35	24
MA	3.19 ± 0.16 ^c^	4.66 ± 0.23 ^c^	0.68	0

Informed values correspond to mean ± standard deviation. Fruit were packed in clamshell (CL), biodegradable films based on corn starch and chitosan (CS:CH), active biodegradable films containing 3% grapefruit seed extract (GSE3) or 3% lemon essential oil (LEO3), and modified atmosphere commercial bags (MA). Different letters within the same column indicate values statistically (*p* < 0.05) different.

**Table 2:** Total phenolic compounds (TPC) and antioxidant capacity (AC) of blueberries var. Emerald stored for 30 days at 1 °C and then submitted 7 days at room temperature.

Sample	TPC (mg CA kg^−1^)	TPC Loss (%)	AC (mg TEAC kg^−1^)	AC Loss (%)
Initial	5765 ± 4.32 ^a^	-	4042 ± 68.82 ^a^	-
CS:CH	4802 ± 73.71 ^b^	16.7	3428 ± 156.54 ^b^	15.2
GSE3	2703 ± 102.65 ^c^	53.1	1827 ± 99.01 ^c^	54.8
CL	5606 ± 122.70 ^a^	2.8	3816 ± 233.73 ^a^	5.6
MA	2091 ± 25.49 ^d^	63.7	1115 ± 29.24 ^d^	72.4

Informed values correspond to mean ± standard deviation. Fruit were packed in clamshell (CL), biodegradable films based on corn starch and chitosan (CS:CH), active biodegradable films containing 3% grapefruit seed extract (GSE3) or 3% lemon essential oil (LEO3), and modified atmosphere commercial bags (MA). Different letters within the same column indicate values statistically (*p* < 0.05) different. TPC and AC losses were calculated with respect to the corresponding initial value.

## Data Availability

Data will be available on request.
